# Incidental Finding of Unilateral Ovarian and Fallopian Tube Agenesis During Cesarean Delivery in Patient With Recurrent Pregnancy Loss

**DOI:** 10.7759/cureus.12769

**Published:** 2021-01-18

**Authors:** Jorge L Alsina, Peter Khamvongsa

**Affiliations:** 1 Obstetrics and Gynecology, Florida International University, Herbert Wertheim College of Medicine, Miami, USA

**Keywords:** partial tubal agenesis, infertility, unilateral ovarian agenesis, ultrasound, cesarean delivery

## Abstract

Congenital unilateral agenesis of the ovary and fallopian tube is a rare condition that has been previously described in the literature. While this condition is benign, studies have proposed it could be associated with infertility. The purpose of this report is first to highlight a rare incidental finding of unilateral ovarian and fallopian tube agenesis. Secondly, we aim to discuss the various imaging modalities used for the detection of uterine, ovarian, and fallopian tube defects and their shortcomings. Our case describes a 37-year-old G4P0030 woman with an obstetric history of spontaneous abortion and ectopic pregnancy, presenting at 38 weeks gestational age with polyhydramnios. The patient received routine obstetric care with no abnormalities being reported on routine ultrasonography. Elective cesarean section was performed at which time the incidental condition discovery of unilateral agenesis of the right ovary and fallopian tube was made. This case is unique since the incidental diagnosis of unilateral right ovarian and right fallopian tube agenesis occurred during cesarean delivery instead of through imaging. It is important for patient counseling to understand the typical workup and deficiencies in pelvic imaging concerning congenital anomalies

## Introduction

Congenital unilateral agenesis of the ovary and fallopian tube is a rare condition that has been previously described in the literature [1]. Incidence has been difficult to determine, but it has been suggested to be around 1:11,240 cases [2]. While most women with this condition present asymptomatically, this anomaly is believed to be associated with infertility [2-4]. The majority of affected patients are diagnosed following laparoscopy or laparotomy for some different cause [1]. Here, we report a case of unilateral agenesis of the fallopian tube and ovary, which was incidentally discovered during cesarean delivery.

## Case presentation

A 37-year-old G4P0030 woman at 38 weeks gestational age was admitted to the labor floor due to polyhydramnios. Vital signs were within normal limits. Her medical history was notable for fibroids and a renal angiomyolipoma. Family history was unremarkable. Obstetric history revealed a history of one elective abortion, one spontaneous abortion occurring at five-weeks gestation age, and a right tubal ectopic pregnancy occurring at six-weeks gestational age. The ectopic pregnancy was treated with methotrexate.

Laboratory tests were all within normal limits. Fetal ultrasound evaluation showed normal fetal growth, with a normal biophysical profile except for her amniotic fluid index, which was 30cm. The non-stress test was reassuring. Considering the patient’s history of prior miscarriages, advanced maternal age, and polyhydramnios, the decision for cesarean delivery was made. Cesarean section was performed in the operating room (OR), and a 3256g healthy male infant with a five-minute Apgar score of eight was delivered.

**Figure 1:**
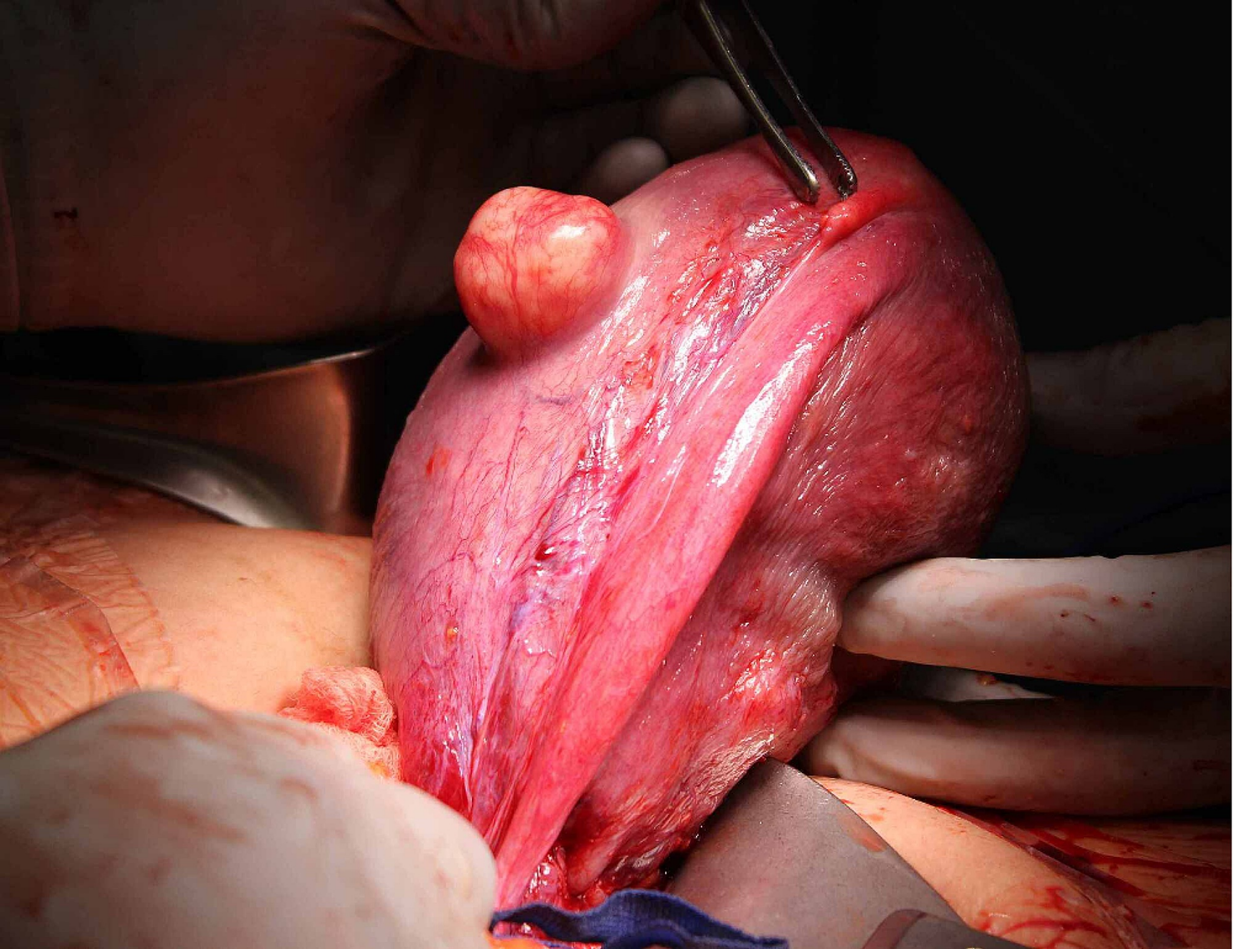
Right-Sided Unilateral Absence of Fallopian Tube (Lateral View)

**Figure 2:**
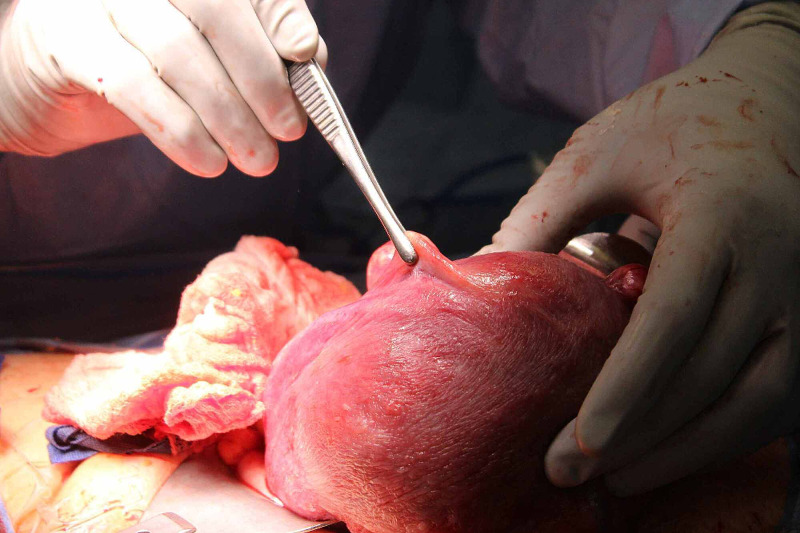
Right-Sided Unilateral Absence of Fallopian Tube (Superior View)

At a cesarean delivery, a small 1cm portion of the fallopian tube at the cornua was identified but the distal segment was absent (Figures 1, 2). In addition, we noticed an absence of the right ovary. In addition, we noticed an absence of the right ovary with retention of the round ligament, parametrial tissue, and appendix. Further inspection displayed, otherwise, normal anatomy on the right side. Our patient reported no prior history of abdominal or pelvic surgery. Prior hysterosalpingogram studies showed obstruction of the right fallopian tube in the midportion. However, at the time these results were interpreted in the context of an ectopic pregnancy. Ultrasonography during her obstetric care failed to visualize any ovarian or fallopian abnormalities. An intravenous pyelogram was not performed; prior renal CT scans were suggestive of no anatomical defects. Her postoperative follow-up was unremarkable except for complaints of anxiety due to our findings. On the third post-operative day, she was dismissed from the hospital with no complications.

## Discussion

**Figure 3:**
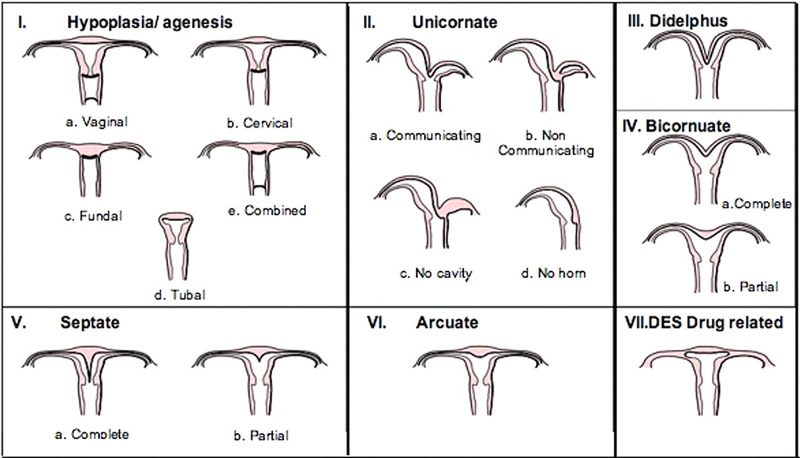
The Classification System of Müllerian Duct Anomalies by the American Fertility Society

Müllerian duct defects represent an uncommon etiology for infertility in women [5]. Studies have estimated the mean prevalence in the general population of fertile, unfertile women, and those suffering recurrent pregnancy loss to be 4.3%, 3.5%, and 13%, respectively [6]. Congenital absence of the ovary and fallopian tube is a rare condition, with a reported incidence of 1:11,240 cases [7,8]. Classification of these abnormalities is thus important. Obstetric outcomes and treatment modalities available differ based on the class of defect [5]. The American Fertility Society in 1988 created a standardized form for the classification of Müllerian defects [8]. This system (Figure 3) relies on a basic understanding of the Müllerian system's embryology and its relation to visual anatomy. Key features for classification include the presence of each segment of the female reproductive tract, the outer contour of the uterine fundus, and the presence of a septum [9]. Mullerian defects and congenital ovarian and fallopian tube agenesis are typically asymptomatic and thus challenging to diagnose.

The number of reported cases remains small but typically, most cases according to the available literature are diagnosed incidentally following laparoscopy for gynecological or obstetric concerns [7]. A few studies even suggest consideration for the ipsilateral absence of the fallopian tube and ovary in the absence of visualization of adnexa on imaging [10]. The identification of these anomalies has historically been done with hysterosalpingography [6]. However, the use of hysterosalpingography only allows for the assessment of the uterine cavity and tubal patency [6]. This limits the clinician’s assessment of the external uterine contour. Ultrasound (US) complements hysterosalpingography by allowing the examination of the external contour. However, ultrasound is not without its pitfalls. Imaging degradation occurs with increased age, BMI, and overflowing bowel gas making the external contour difficult to visualize [6,11].

Additionally, visualization of the ovaries and fallopian tubes can present an added challenge. While most ultrasound specialists can identify these structures, it is not unheard of for them to be missed. One study by Lefringhouse et al. examined the accuracy of detection in normal women with transvaginal ultrasound. They found that ovaries were observable in 82.7% of cases while the fallopian tubes were observable in 77.2%, increasing to 85.2% if the ovary was identifiable [12]. Various studies have also reported on the challenges of detecting fallopian tubes in the absence of pathology (thickened, inflamed, and edematous) [12]. What makes this even more challenging is that absence does not necessarily equal disease. One study by Motuzko et al. showed that the absence of detection of the ovary on pelvic CT or US was highly predictive for lack of ovarian abnormality on short-term follow-up and did not require additional clinical imaging to exclude ovarian disease [2]. Despite these reported challenges and the limited literature available on congenital agenesis of the ovary and fallopian tube, most cases follow a clinical workup with hysterosalpingography showing occlusion of the affected fallopian tube and ultrasound showing an absent ovary [13].

## Conclusions

Our case was interesting in that the patient reports never having received any abnormal ultrasound findings during her obstetric care. To our best knowledge, this case is unique since the incidental diagnosis of unilateral right ovarian and right fallopian tube agenesis occurred during Cesarean delivery. It is important for patient counseling to understand the typical workup and deficiencies in pelvic imaging concerning congenital anomalies. While this condition is benign, this incidental discovery can have a stressful psychological impact on the patient. Thus, awareness of this rare condition can be helpful information for the clinician to consider.
